# Effect of ketotifen premedication on adverse reactions during peanut oral immunotherapy

**DOI:** 10.1186/1710-1492-10-36

**Published:** 2014-07-09

**Authors:** Amanda Jagdis, Noam Berlin, Carly Barron, Mohana Giruparajah, Nathan Leader, Sean Maclachlan, Gordon L Sussman

**Affiliations:** 1 Division of Clinical Immunology and Allergy, Department of Medicine, University of Toronto, 202 St Clair Ave W, Toronto, ON M4V 1R2, Canada; 2 Faculty of Medicine, University of Toronto, Toronto, ON, Canada; 3 Gordon Sussman Clinical Research Inc., Toronto, ON, Canada; 4 Melbourne Medical School, University of Melbourne, Melbourne, Australia; 5 Faculty of Medicine, University of Ottawa, Ottawa, ON, Canada

**Keywords:** Peanut allergy, Oral immunotherapy, Antihistamine premedication, Ketotifen, Desensitization, Adverse reactions

## Abstract

**Background:**

Oral immunotherapy (OIT) has shown promise in inducing desensitization for food allergy. However, there are safety concerns regarding the frequency and severity of adverse events during food OIT.

**Objective:**

To evaluate the effect of Ketotifen premedication on adverse reactions during peanut OIT.

**Methods:**

A randomized single blind placebo controlled pilot study was performed. Peanut OIT was performed using a previously published protocol. Ketotifen was up-titrated to 2 mg twice daily over two weeks (week -2 to 0), followed by a peanut OIT initial escalation day (day 1). Ketotifen was administered from week 0–4 of peanut OIT; reactions to peanut OIT doses were recorded by clinic staff and subject diary.

**Results:**

Six subjects (median age 10 years, peanut IgE >100kU_A_/L) were enrolled, 4 randomized to Ketotifen, 2 to placebo. The most common side effect of Ketotifen was fatigue (9% during up-titration). The rate of reaction per peanut OIT dose was lower for subjects on ketotifen (K) compared to placebo (P) during initial escalation on day 1 (K: 22% (8/36) vs. P: 67% (12/18)); week 0–4 build-up doses (K: 75% (3/4) vs. P: 100% (2/2)); and week 0–4 home doses (K: 50% (54/108) vs. P: 82% (27/33)). The rate of gastrointestinal symptoms per peanut OIT dose was also lower for subjects on ketotifen during initial escalation on day 1 (K: 17% (6/36) vs. P: 61% (11/18)); week 0–4 build-up doses (K: 75% (3/4) vs P: 100% (2/2)); and week 0–4 home doses (K: 46% (50/108) vs. P: 82% (27/33)).

**Conclusions:**

Ketotifen premedication is well tolerated and reduces the rate of gastrointestinal symptoms during peanut OIT. These findings require confirmation in a larger study of Ketotifen premedication used throughout peanut OIT.

**Trial registration:**

Clinical Trials number: NCT0162515

## Background

Peanut allergy is a significant health concern in North America: an estimated 1.4% of children in the United States have a diagnosis of peanut allergy, and the prevalence is rising [1]. Peanut allergy generally presents in childhood, is persistent in approximately 80% of cases, [2,3] and is the most commonly implicated food allergy in fatal anaphylactic reactions [4]. There is currently no cure for peanut allergy. Management of peanut allergy involves strict avoidance of peanut and carriage of self-injectable epinephrine for emergency treatment, should an accidental ingestion occur. The ongoing need for diligent avoidance and the fear of accidental ingestion has a significant impact on patients’ quality of life [5].

The development of active approaches to treatment of peanut allergy is underway. Early study of subcutaneous immunotherapy in food allergy was stopped due to frequent systemic reactions [6,7]. Oral immunotherapy for food allergy is now being evaluated. Early studies of oral immunotherapy (OIT) for peanut allergy have shown promise in inducing desensitization [8,9]. However, further investigation into the safety and efficacy profile is needed before peanut OIT can be recommended for clinical use [8,10]. Frequent and sometimes severe adverse allergic reactions have been observed during peanut OIT. In a recent systematic review, adverse reactions were reported in all six studies examined, with the highest frequency of reactions during periods of rapid dose escalation [8]. A study of the safety profile of peanut OIT reported that 93% of patients experienced adverse reactions during the initial peanut dose escalation day, involving predominantly gastrointestinal, upper respiratory, and cutaneous symptoms [11].

The issue of adverse reactions during immunotherapy is not limited to peanut OIT. Immunotherapy for hymenoptera venom allergy is highly effective, but associated with frequent and occasionally severe adverse allergic reactions, particularly during the dose escalation phase. Use of antihistamine premedication has been demonstrated to reduce local and systemic reactions during venom immunotherapy, without affecting the efficacy of venom immunotherapy [12,13]. The mechanism is thought to involve alteration in histamine receptor expression on allergen-specific T cells and altered cytokine profiles observed in patients receiving premedication with antihistamines [13].

Ketotifen is a benzocycloheptathiophene derivative with histamine receptor antagonist and mast cell stabilizing properties [14]. Clinically, ketotifen has been previously used for conditions ranging from childhood asthma to eosinophilic gastroenteritis, urticaria pigmentosa, and cold urticaria [15-18]. Ketotifen has demonstrated efficacy in treatment of allergic conjunctivitis, although is less effective and comfortable compared to ophthalmic olopatadine [19,20]. Ketotifen was also investigated for use in pediatric systemic mastocytosis, but in comparison to hydroxyzine, offered no symptomatic benefit or change in plasma or 24-hour urine histamine levels [21]. At present, ketotifen is indicated for treatment of allergic conjunctivitis, and is available in ophthalmic formulation only in the USA. Oral ketotifen is available in Canada, and is considered a safe medication, the dose of which is limited by the sedating side effects [22].

The aim of this study was to evaluate the use of premedication in the setting of oral immunotherapy for peanut allergy. Ketotifen was selected as the premedication agent for its dual antihistamine and mast cell stabilizing properties; established safety record; and, to examine whether it may mitigate gastrointestinal symptoms frequently observed during food oral immunotherapy given its previously documented benefit in eosinophilic gastroenteritis. Using a previously published protocol for peanut OIT [9,23] we conducted a small randomized, single-blind, placebo controlled pilot study examining the rate and severity of adverse reactions during peanut OIT with ketotifen premedication during the initial four weeks of peanut dose escalation.

## Methods

### Subject recruitment and selection

Forty subjects were screened from this Allergy & Immunology clinic. The accrual objective was six subjects for this pilot study. Inclusion criteria included: age ≥ 8 years; peanut skin prick test ≥ 3 mm, and peanut specific IgE >7 kU_A_/L (CAP-FEIA, Phadia AB; Pharmacia, Inc, Uppsala, Sweden); and convincing history of reaction within 60 minutes of peanut consumption. Exclusion criteria were: moderate/severe persistent asthma; current requirement for greater than medium daily doses of inhaled corticosteroids, as defined by the NHLBI guidelines; poorly controlled or persistent atopic dermatitis; diabetes; known oat or wheat allergy (due to potential cross-contamination); severe anaphylaxis to peanut as defined by hypoxia, hypotension, or neurological compromise; inability to discontinue antihistamines for skin testing; history of epilepsy or seizures; current participation in an investigational drug study; or participation in an interventional treatment for food allergy within the previous 12 months.

This study was performed with approval by Canadian Shield Ethics Board, and written informed consent was obtained from subjects and their guardian. Other than peanut OIT, subjects continued strict avoidance of peanut in their diet prior and during the study*.*

### Randomization

Randomization was performed by use of blinded envelopes in a ratio of 2:1 for ketotifen: placebo. Randomization allocation was stored in a locked database accessible only by clinical staff. Clinical staff were aware of assigned intervention; subjects remained blinded until completion of the study.

### Study design

**Figure 1:**
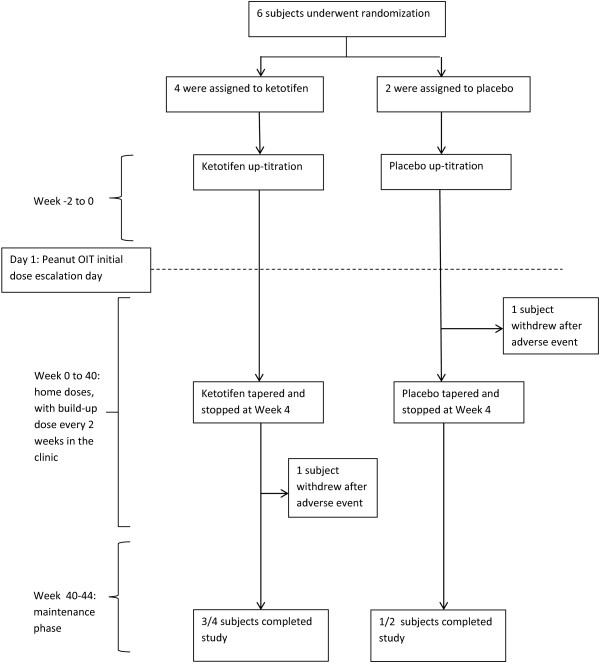
Study design.

This was a single-blind placebo-controlled study (Figure 1). During the initial visit, a baseline history, physical exam, and blood draw for peanut specific IgE was performed. After randomization subjects underwent a 2 week up-titration of ketotifen dosing or placebo, up to a maximum dose of 2 mg ketotifen twice daily. This dose was approved by Health Canada and chosen to maximize efficacy of the premedication protocol. This was followed by an initial day escalation phase for peanut OIT, long term build-up phase over 44 weeks, and maintenance phase for 4 weeks at the highest tolerated dose, using a previously described protocol modified for use in the outpatient clinic [9,23]. The ketotifen dose was tapered and then discontinued at 4 weeks after the initial escalation day based on IRB approval. The primary endpoint for this study was the rate and severity of adverse reactions occurring on the initial escalation day in the ketotifen group compared to the placebo group. The secondary endpoints for the study included: the rate and severity of gastrointestinal adverse reactions, the overall rate of adverse reactions throughout the OIT protocol, and the rate of adverse reactions leading to study withdrawal in the ketotifen and placebo groups, respectively.

### Skin prick testing

Skin prick testing was performed prior to enrollment with commercial peanut extract, histamine and saline controls (Alk-Abello, Ontario, Canada). Skin tests were read after 15 minutes, and the mean wheal diameter was recorded.

### Ketotifen dosing

Ketotifen (Novopharm, Toronto, Canada) was initiated and up-titrated as follows: 1 mg daily for 3 days, 1 mg twice daily for 4 days, 2 mg twice daily for 7 days, followed by the initial escalation day. Subjects continued ketotifen 2 mg twice daily on the initial escalation day. Following the initial escalation day, ketotifen was maintained at 2 mg twice daily for 2 weeks, then reduced to 1 mg twice daily for 2 weeks, then stopped at day +28, based on IRB approval. If subjects reported side effects such as drowsiness on ketotifen, the dose was either reduced to the highest previously tolerated dose, or maintained without dose escalation. Placebo tablets (250 mg lactose tablets, Odan, Montreal, Canada) were administered in the same manner.

### Peanut flour

Premeasured peanut flour (from Partially Defatted Peanut Flour 12% Fat Light Roast; Golden Peanut Company, Alpharetta, Ga; 2 g flour = 1 g peanut protein) doses were provided to subjects in capsule form. Capsules were taken intact, not mixed with food, either with or without a meal. Health Canada approval was obtained for use of this peanut flour capsule in the clinical trial setting.

### Initial escalation day

The initial escalation day consisted of a one-day (12 hour) rapid dose escalation, performed using protocol published previously by Burks *et al.*[23] slightly modified for the outpatient setting. Peanut flour capsules were administered orally from a starting dose of 0.1 mg peanut protein in 30 minute intervals until a maximum 25 mg of peanut protein was tolerated. The maximum dose was modified to 25 mg rather than 50 mg of peanut protein as the protocol was performed in an outpatient clinic setting. Epinephrine, diphenhydramine, cetirizine and salbutamol were kept at the bedside. An intravenous catheter was placed prophylactically, vital signs were measured every 30 minutes and subjects were evaluated by the study investigator before each dose escalation was permitted.

### Home dosing

Subjects were instructed to take peanut flour doses daily with food, and were provided with pre-measured capsules. Subjects were instructed to contact the clinic for dosing instruction with febrile illness or missed doses.

### Build-up visits

The build-up phase was performed using a previously published protocol, again slightly modified for the outpatient setting [9]. Subjects returned to the clinic for dose escalations every two weeks for 44 weeks. The dose was increased by 50% at the first visit (total 50 mg peanut protein), followed by dose increases of 14-33% thereafter, until a maximum daily maintenance dose of 4000 mg peanut protein was reached.

### Maintenance phase

At week 44, subjects continued on 4000 mg peanut protein daily, or their highest tolerated dose, as a maintenance dose for 4 additional weeks.

### Safety

Subjects were instructed to keep a daily symptom log for the duration of the study. Subjects were evaluated for symptoms and adverse reactions based on daily symptom logs every 2 weeks in the clinic. Reaction symptoms observed in the clinic were graded as 1, 2, or 3, based on the classification scheme previously published by Brown [1]. Reaction symptoms from patient diaries were defined as follows; mild: transient and easily tolerated; moderate: caused discomfort and interrupted the subject’s usual activities; severe: caused considerable interference with the subject’s usual activities, was incapacitating or life-threatening. Anaphylactic reactions were defined as per the definition of anaphylaxis published by Sampson *et al.* in 2006 [24].

## Results

### Study population

Forty subjects were screened from a list of peanut-sensitized children followed at this outpatient clinic. Seven children and their parents met inclusion criteria and consented to participate, one subject withdrew prior to the start the study for a final enrollment of 6 subjects. Four subjects were assigned to receive ketotifen premedication, 2 subjects were assigned to receive placebo. The median age and baseline clinical characteristics were similar in each group (Table 1).

**Table 1:** Baseline clinical characteristics

**Clinical characteristic**	**Ketotifen group**	**Placebo group**
Number	4	2
Median Age (years)	10	10
Sex	1 female, 3 male	1 female, 1 male
History of asthma	2/4	1/2
History of allergic rhinitis	1/4	0/2
History of atopic dermatitis	2/4	0/2
History of other food allergy	1/4	1/2
Initial median size (mm) of peanut skin prick test, (range)	10 (7–16)	15 (15)
Initial median peanut specific IgE (kU_A_/L), (range)	>100 kU/L (44.9 - >100)	>100 kU/L (>100)

### Ketotifen titration

Ketotifen subjects received a total of 53 days of ketotifen up-titration and placebo subjects received 28 days of placebo prior to the initial escalation day. No serious adverse events related to ketotifen were observed. Fatigue was the major adverse effect noted in the ketotifen group, occurring only at the highest dose of 2 mg BID, on 5/25 days (20%) at this dose. Adverse symptoms reported during up-titration of ketotifen (K) versus placebo (P) included: fatigue (K: 5 days/53 days, 9%) vs. (P: 0 days/28 days); gastrointestinal symptoms (K: 6 days/53 days, 11%) vs. (P: 4 days/28 days, 14%); upper respiratory tract infection (URTI) (K: 2 days/53 days, 4%) vs. (P: 2 days/28 days, 7%); along with ongoing pre-existing seasonal rhinitis and atopic dermatitis. Headache (2 days/53 days, 4%) and blurred vision with dizziness (1 days/53 days, 2%) occurred in one ketotifen subject and required temporary delay in up-titration. At the time of initial escalation day, all subjects had received 11 to 15 days of ketotifen premedication and were taking ketotifen 2 mg twice daily. In the four weeks following the initial escalation day, adverse effects of ketotifen was difficult to interpret, as most adverse effects were related to peanut OIT. Fatigue, however, was reported in ketotifen subjects on 4 days/118 days (3%), and no placebo subjects. Compliance with ketotifen doses was estimated at 92% (148 tablets taken/161 tablets) prior to initiation of peanut OIT and 99% (392 tablets taken/394 tablets) during the first four weeks of peanut OIT. One subject remained on ketotifen for a total of 50 days as they were unable to attend the clinic at the regular visit time. One subject accidentally continued ketotifen 2 mg bid for 3 weeks, rather than 2 weeks following initial escalation day, then subsequently reduced the dose to 1 mg bid for the last week of ketotifen treatment.

### Initial escalation day

The maximum dose of 25 mg peanut protein was reached by all subjects in the ketotifen and placebo groups. Clinically relevant symptoms, defined as symptoms suggestive of an adverse allergic reaction, occurred in 3 of 4 ketotifen subjects and both placebo subjects (Table 2). Symptoms were elicited at doses between 0.05 and 25 mg of peanut protein.

**Table 2:** Adverse reactions to peanut OIT on initial escalation day (Day 1)

**Adverse reactions and treatment**	**Ketotifen**	**Placebo**
Total no. of doses of OIT, n	36	18
Total no. of patients reporting symptoms	3/4	2/2
Total no. of doses with symptoms, n (% of total OIT doses)	8/36 (22%)	12/18 (67%)
Anaphylaxis, n (% of total OIT doses)	-*	1 (5%)
**Symptoms n, (% of OIT doses)**	**Ketotifen**	**Placebo**
Gastrointestinal	6/36 (17%)	11/18 (61%)
Cutaneous	-	1/18 (6%)
Lower respiratory	2/36 (6%)	1/18 (6%)
Oropharyngeal	-	2/18 (11%)
Upper respiratory	-	-
Cardiovascular	-	-
Other^A^	-	2/18 (11%)
**Treatment n, (% of OIT doses)**	**Ketotifen**	**Placebo**
Epinephrine	-	-
Diphenhydramine	-	-
Cetirizine or other**	-	1/18 (6%)
Prednisone	-	-
Salbutamol or other inhaled medication	-	1/18 (6%)

*(-) indicates nil reported.

^A^other symptoms reported: Grade 1 headache.

**Other second generation antihistamine.

Ketotifen subjects had a lower rate of reaction than placebo subjects on the initial escalation day. Ketotifen subjects experienced a lower rate and lower grade of gastrointestinal symptoms (6/36 doses, 17%, all Grade 1) than placebo subjects (11/18 doses, 61%; 2/18 Grade 1; 6/18 Grade 2; 3/18 Grade 3). The rate of cutaneous, lower respiratory and oropharyngeal symptoms was similar in both groups, and none were of Grade 3 severity. Other symptoms were reported in placebo patients only and one episode of anaphylaxis occurred in the placebo group. Treatment with antihistamines and inhaled beta agonists was required in the placebo group only.

### Build-up and home doses while on ketotifen (week 0–4)

Both ketotifen and placebo subjects experienced symptoms, predominantly gastrointestinal, following most peanut OIT build up doses (Table 3). The rate of gastrointestinal symptoms was similar in both groups. The only other symptoms reported were cutaneous symptoms in placebo subjects. Treatment with antihistamines was required in the ketotifen group only.

**Table 3:** Adverse reactions to peanut OIT while on Ketotifen (day 2-week 4)

**Adverse reactions and treatment**	**Build-up doses in clinic**		**Home doses**	
	**Ketotifen**	**Placebo**	**Ketotifen**	**Placebo**
Total no. of doses of OIT, n	4	2	108	33
Total no. of missed OIT doses, n	-	-	-	-
Total no. of patients reporting symptoms	3	1	4	2
Total no. of doses with symptoms, n (% of OIT doses)	3/4 (75%)	2/2 (100%)	54/108 (50%)	27/33 (82%)
Anaphylaxis, n (% of OIT doses)	-	-	1/108 (1%)	1/33 (3%)
**Symptoms n, (% of OIT doses)**	**Ketotifen**	**Placebo**	**Ketotifen**	**Placebo**
Gastrointestinal	3/4 (75%)	2/2 (100%)	50/108 (46%)	27/33 (82%)
Cutaneous	-	1/2 (50%)	1/108 (1%)	6/33 (18%)
Lower respiratory	-	-	6/108 (6%)	2/33 (6%)
Upper respiratory	-	-	2/108 (2%)	-
Oropharyngeal	-	-	1/108 (1%)	-
Cardiovascular	-	-	-	-
Other	-	-	14/108 (13%)^B^	2/33 (6%)^B^
**Treatment n, (% of OIT doses)**	**Ketotifen**	**Placebo**	**Ketotifen**	**Placebo**
Epinephrine	-	-	-	1/33 (3%)
Diphenhydramine	-	-	1/108 (1%)	1/33 (3%)
Cetirizine**	1/4 (25%)	-	7/108 (6%)	5/33 (15%)
Prednisone	-	-	-	1/33 (3%)
Salbutamol or other inhaled medication	-	-	1/108 (1%)	-

^B^Other reported symptoms included: fever, headache, fatigue, pallor.

**Other second generation antihistamine.

Ketotifen subjects had a lower rate of reaction than placebo subjects after peanut OIT home doses (Table 3). No peanut OIT home doses were missed among either group. Ketotifen subjects experienced a lower rate of gastrointestinal symptoms (50/108 doses, 46%) than placebo subjects (27/33 doses, 82%). Ketotifen subjects also experienced a lower rate of cutaneous symptoms (1/108 doses, 1%) than placebo subjects (6/33 doses, 18%). The rate of lower respiratory, upper respiratory, and oropharyngeal symptoms was similar in both groups. Anaphylaxis occurred after home dosing in 1/108 doses (1%) and 1/33 doses (3%) in ketotifen and placebo patients, respectively. Treatment for adverse reactions was required in both the ketotifen (antihistamine and inhaled beta agonist) and placebo groups (epinephrine, antihistamine, and prednisone).

### Build-up, home doses and maintenance off ketotifen (week 4–44)

Ketotifen subjects had a lower rate of rate of reaction than placebo subjects following peanut OIT build-up doses (Table 4). Ketotifen subjects reported a lower rate of gastrointestinal symptoms (9/61 doses, 15%) than placebo subjects (15/19 doses, 79%) and a lower rate of respiratory symptoms (2/61 doses, 3%) than placebo subjects (2/19 doses, 11%). Ketotifen subjects reported a higher rate of upper respiratory symptoms (4/61 doses, 6%) than placebo subjects (0/19 doses). The rate of cutaneous and oropharyngeal symptoms was similar, with no anaphylactic reactions and no treatment required in either group.

**Table 4:** Adverse reactions to peanut OIT off ketotifen (week 4–44)

**Adverse reactions and treatment**	**Build-up doses in clinic**		**Home doses**	
	**Ketotifen**	**Placebo**	**Ketotifen**	**Placebo**^**C**^
Total no. of doses of OIT, n	61	19	1221	373
Total no. of missed OIT doses, n	-	-	1/1221 (.08%)	-
Total no. of doses with symptoms, n (% of total OIT doses)	19/61 (31%)	15/19 (79%)	156/1221 (13%)	97/373 (26%)
Anaphylaxis, n (% of total OIT doses)	-	-	5/1221 (0.4%)	-
**Symptoms n, (% of OIT doses)**	**Ketotifen**	**Placebo**	**Ketotifen**	**Placebo**
Gastrointestinal	9/61 (15%)	15/19 (79%)	95/1221 (8%)	83/373 (22%)
Cutaneous	-	-	12/1221 (0.9%)	15/373 (4%)
Lower respiratory	2/61 (3%)	2/19 (11%)	23/1221 (2%)	12/373 (3%)
Upper respiratory	4/61 (6%)	-	55/1221 (5%)	-
Oropharyngeal	2/61 (3%)	-	12/1221 (0.9%)	-
Cardiovascular	-	-	-	-
Other	8/61 (13%)	-	122/1221 (10%)^D^	12/373 (3%)^D^
**Treatment n, (% of OIT doses)**	**Ketotifen**	**Placebo**	**Ketotifen**	**Placebo**
Epinephrine	-	-	1/1221 (0.08%)	-
Diphenhydramine	-	-	11/1221 (0.9%)	-
Cetirizine**	-	-	44/1221 (4%)	1/373 (0.2%)
Prednisone	-	-	2/1221 (0.1%)	-
Salbutamol or other inhaled medication	-	-	27/1221 (2%)	9/373 (2%)

^C^One placebo subject withdrew during the initial 4 weeks of peanut OIT and therefore was not included in these results.

^D^Other reported symptoms/adverse effects included: atopic dermatitis, headache, gastroenteritis, pinworms, seasonal allergic rhinitis, strep throat and URTI.

**Other second generation antihistamine.

Ketotifen subjects had a lower rate of reaction than placebo subjects following peanut OIT home doses (Table 4). One peanut OIT home dose was missed in a ketotifen subject. Ketotifen subjects reported a lower rate of gastrointestinal symptoms (95/1221 doses, 8%) than placebo subjects (83/373 doses, 22%) and a higher rate of upper respiratory symptoms (55/1221 doses, 5%) than placebo subjects (0/373 doses). The rate of cutaneous, lower respiratory and oropharyngeal symptoms was similar. Anaphylaxis occurred after home dosing in 5/1221 (0.4%) of ketotifen subjects, and no placebo subjects. Treatment for adverse reactions was required in both the ketotifen (epinephrine, antihistamine, prednisone, inhaled beta agonists) and placebo groups (antihistamine, inhaled beta agonists).

### Anaphylaxis

In total, 8 episodes of anaphylaxis were reported, 6 in ketotifen subjects and 2 in placebo subjects. The overall incidence of anaphylaxis per dose of peanut OIT was 6/1430 (0.4%) in ketotifen subjects and 2/445 (0.4%) for placebo subjects. Epinephrine was used for treatment of only two anaphylactic reactions. Symptoms of anaphylaxis included: cutaneous (8/8), lower respiratory (7/8), upper respiratory (3/8), oropharyngeal (6/8), gastrointestinal (5/8), cardiovascular (0/8). Anaphylaxis after home doses occurred in the presence of known cofactors including: fever/infection (2/7), exercise (4/7) and menstruation (1/7).

### Study withdrawal

One subject withdrew prior to the start of the study and therefore was not included in the analysis. One placebo subject withdrew from the study on day 7, and one ketotifen subject withdrew in week 36, both following an anaphylactic reactions requiring emergency room treatment.

### Final dose and peanut specific IgE

Three ketotifen subjects and one placebo subject completed the 44 week peanut OIT protocol. The median dose of peanut protein that elicited symptoms on initial escalation day was 200 mcg. For subjects completing peanut OIT, the median dose of peanut protein tolerated was 1825 mg for both the ketotifen and placebo subject(s). Subjects were then dose adjusted to receive ongoing maintenance with either whole roasted peanut, or peanut M&M’s^®^, per subject preference. The first dose of whole peanut or M&M’s^®^ was administered in the clinic in a monitored setting. The median peanut specific IgE was 84 kU_A_/L at completion of peanut OIT (median 69 kU_A_/L in ketotifen subjects; >100 kU_A_/L in the placebo subject).

## Discussion

Although early studies of peanut OIT appear to show promise in inducing desensitization [8,9], the frequency and severity of adverse allergic reactions during peanut OIT remain a concern. The highest reported rates of adverse allergic reactions in peanut OIT have typically been during periods of rapid dose escalation [8]. In this small proof of concept study, we examined the use of ketotifen premedication during the initial 4 weeks of peanut OIT.

The present study was limited by the small number of patients and short duration. Statistical analysis was not feasible given the small numbers, which prevents us from drawing firm conclusions. However, this work was performed as a pilot study, and requires confirmation in a larger scale study of ketotifen premedication used throughout OIT.

All subjects experienced clinically relevant adverse symptoms at some point during peanut OIT, in keeping with other reports of peanut OIT using similar protocols [9,23]. Subjects receiving ketotifen premedication had a lower frequency and severity of reactions on the initial escalation day and during the first four weeks of peanut OIT than subjects receiving placebo. This was in large part due to a reduction in gastrointestinal reactions. Gastrointestinal (GI) symptoms were the most frequently reported clinically relevant symptoms on the initial escalation day and, in fact, throughout the duration of peanut OIT. In contrast, in a previous safety study of a similar peanut OIT regimen, upper respiratory symptoms were the most common, and gastrointestinal symptoms were considerably less frequent during home doses [11]. A lower overall frequency of GI symptoms and, in particular, a lower frequency of moderate to severe GI symptoms was observed in subjects receiving ketotifen premedication. In eosinophilic gastroenteritis, which may not be analogous to food OIT, ketotifen treatment has been associated with symptomatic and histologic improvement [16]. The postulated mechanisms behind its efficacy include H1-antihistamine activity, stabilization of mast cells, and possibly impairment of eosinophil migration [16,25]. In our study, the trend of reduced GI symptoms appeared to persist in the ketotifen subjects after ketotifen was discontinued. There is one case report describing prolonged remission of eosinophilic gastroenteritis after one month of ketotifen treatment [25]. However, given the small number of study subjects, individual variation in susceptibility to GI symptoms, viral gastroenteritis and other causes of transient GI upset are likely to have influenced our results.

Lower respiratory symptoms were experienced at similar frequency between both groups during the initial escalation day, home doses and build-up doses. Asthmatic subjects were included in both groups, and moderate to severe lower respiratory symptoms were rare. Ketotifen is known to have a delayed onset of action in asthma, taking up to 6–12 weeks to achieve maximal response [14]. Therefore our six week trial of ketotifen may have been insufficient to detect any effect on lower respiratory reactions, particularly during the initial dose escalation day which was performed after two weeks of ketotifen premedication. In addition, the small number of subjects and the relative infrequency of lower respiratory reactions would make it difficult to detect a difference.

Upper respiratory symptoms were predominantly reported with home doses after ketotifen was discontinued, and therefore may have initially been suppressed by the H1-antihistamine activity of ketotifen. However, these results were also likely influenced by other common causes of transient upper respiratory symptoms such as viral infection and sinusitis. One ketotifen subject had allergic rhinitis prior to starting peanut OIT and required treatment with Cetirizine for approximately seven weeks after ketotifen was discontinued which likely influenced the adverse effects reported by this patient. Oropharyngeal symptoms were relatively uncommon in both groups, and no cardiovascular symptoms were reported.

Cutaneous symptoms were rarely reported while on ketotifen premedication, again suggesting suppression by the H1 antihistamine activity of ketotifen. This raises the issue of potential “masking” or partial treatment of a reaction by ketotifen premedication leading to under-recognition of a reaction by patients or their parents. This is concerning as many patients recognize cutaneous symptoms as key initial symptoms of reaction. Additional safety studies are required before premedication can be recommended during oral immunotherapy for food allergy. However, if premedication is used in future OIT research, patients should be educated on recognition of atypical presentations of anaphylaxis.

Eight episodes of anaphylaxis occurred, all involving cutaneous symptoms, and the majority occurring after home doses taken in the context of known cofactors of anaphylaxis. As a result of anaphylactic reactions, one subject from each of the ketotifen and placebo groups withdrew from the study. Accidental peanut ingestion and accidental overdose of peanut OIT were not observed but remain relevant concerns. Despite education, epinephrine was used in only two cases of anaphylaxis. The ongoing risk of anaphylaxis with home doses of peanut OIT should be emphasized to patients, particularly in the setting of cofactors of anaphylaxis, and patients should be prepared to treat reactions should they occur.

As ketotifen was continued for only the first four weeks of OIT, we cannot predict whether ongoing ketotifen premedication would have reduced frequency and severity of adverse reactions experienced during the remainder of peanut OIT. For the same reason, we cannot predict whether the use of ketotifen premedication would have influenced the efficacy of peanut OIT. At the end of the study, the remaining placebo and ketotifen patient(s) tolerated equivalent median doses of peanut protein.

Oral immunotherapy is a promising area of investigation for treatment of peanut and other food allergy. However, safety concerns remain regarding the frequency and severity of adverse reactions during peanut OIT, particularly during periods of rapid dose escalation [8]. Our findings suggest that ketotifen premedication is well tolerated and reduces frequency and severity of gastrointestinal adverse reactions during peanut OIT. Larger randomized studies of the safety and efficacy of premedication during oral immunotherapy for food allergy are warranted.

## Abbreviations

OIT: Oral immunotherapy.

## Competing interests

The authors declare that they have no competing interests.

## Authors’ contributions

AJ wrote the manuscript. GS conceived of the study, participated in the design and coordination of the study, and revised the manuscript. NB, MG, NL and SM, participated in coordination of the study and data collection. CB participated in data collection. All authors read and approved the final manuscript.
